# Synuclein phosphorylation: pathogenic or physiologic?

**DOI:** 10.1038/s41531-023-00487-z

**Published:** 2023-03-28

**Authors:** Christiana Kontaxi, Robert H. Edwards

**Affiliations:** UCSF School of Medicine, Departments of Neurology and Physiology, San Francisco, USA

**Keywords:** Diseases of the nervous system, Neurophysiology

Despite multiple therapeutic avenues for the management of symptoms in Parkinson’s disease (PD), we still lack treatment for the underlying degenerative process. As a result, the disease continues to progress, becoming refractory to treatment and resulting in severe disability. To address this, investigators have focused on the proteins implicated in PD by human genetics and in particular on the protein α-synuclein, which accumulates in the nervous system of patients with PD and the related disorders dementia with Lewy bodies (LBD) and multiple system atrophy (MSA). The misfolding of synuclein and the dominant inheritance of PD due to mutations in synuclein and leucine rich repeat kinase 2 (LRRK2) have led to the hypothesis that the disease involves a gain of abnormal function. As a corollary, the normal function of these proteins has been considered irrelevant for disease. It is true that loss of synuclein does not cause PD: knockout mice lacking all three synuclein isoforms do not show either cell loss or parkinsonian motor symptoms^1,2^. However, degeneration arises in the context of normal function, making it essential to understand the adaptive, physiological role of these proteins, which has remained elusive.

Dettmer, Selkoe and colleagues now address the physiological role of a post-translational modification associated with the synuclein^+^ Lewy pathology characteristic of PD^3^. Considered specific for PD, phosphorylation of α-synuclein on Ser-129 (pSer-129)^4^ has been widely used to assess the magnitude and extent of degeneration in animal models as well as the human condition. In general, it has been considered a marker of Lewy pathology, and the role of pSer-129 in pathogenesis has been the subject of debate. Several reports implicated the modification in toxicity^5–7^ but another suggested an inhibitory effect on synuclein fibrillization^8^. And other studies found no effect of the modification at all^9–11^. However, none of these studies focused on a potential physiological role for pSer-129, although previous work had detected this modification in normal brain with some regulation by a strong sensory stimulus^12,13^. In addition, this residue is highly conserved in α-synuclein but not β- or γ- isoforms, suggesting a specific, adaptive role.

In the current study, Ramalingam et al. show that neural activity increases pSer-129 ~3-fold in primary culture, with no change in total α-synuclein^3^. This induction requires action potentials and synaptic transmission, indicating that network activity is responsible. The authors also observe a more modest increase in brain extracts after environmental enrichment, supporting the relevance of activity in pSer-129 induction in vivo. In addition, the activity-dependence of phosphorylation appears specific for Ser129, not other known sites of phosphorylation in α-synuclein. To understand how activity increases pSer-129, the authors then identify polo-like kinase 2 (Plk2) as responsible. Ca^++^ often mediates the effect of activity and the authors find that voltage-gated Ca^++^ channels are important for the phosphorylation, but Plk2 is not known to respond to Ca^++^. The authors thus sought a potential upstream activator, identifying the Ca^++^-sensitive phosphatase calcineurin. Inhibition of calcineurin reduces pSer-129 to the same extent as inhibition of Plk2, with no additive effects, suggesting they act in the same pathway. Importantly, calcineurin has a major role in neurotransmitter release and α-synuclein is highly presynaptic.

To determine where in the cell Ser-129 phosphorylation occurs, the authors fractionate neurons after the induction of neural activity and find that most (but not all) of the modified α-synuclein associates with membranes. Immunostaining confirms the expression of pSer-129 at presynaptic boutons. However, perhaps the most remarkable result is the increased phosphorylation in vitro with Plk2 in the presence of liposomes. α-Synuclein is a peripheral membrane protein that probably associates with synaptic vesicles and membrane association with artificial membranes in vitro requires the highly conserved N-terminus, with seven 11-amino acid repeats that form an α-helix on membrane binding^14^. pSer-129 occurs at the less highly conserved C-terminus, and the increase in Ser-129 phosphorylation on membrane association may occur by displacement of the C-terminus from an intramolecular interaction with the N-terminal membrane-binding repeats. Membrane association may similarly influence binding of the α-synuclein C-terminus to v-SNARE VAMP2^1^. Since α-synuclein binds with low affinity to presynaptic membranes and cycles dynamically on and off with exocytosis and recycling^15^, membrane association as well as the activation of Plk by calcineurin may regulate Ser-129 phosphorylation. However, phosphorylation at Ser-129 does not appear to influence the interaction of α-synuclein with membranes, so membrane association appears to promote phosphorylation at Ser-129 rather than vice versa. This presumably accounts for the increased presynaptic localization of synuclein phosphorylated at Ser-129 that the authors also demonstrate.

Finally, Ramalingam et al. (2023) address the functional role of Ser-129 phosphorylation^3^, a tall order given the unclear role of α-synuclein in neurotransmission studied in KO mice^1,2^. Introducing WT and mutant α-synuclein that cannot undergo phosphorylation at Ser-129 into α-synuclein KO neurons, they find that relative to WT, the mutant reduces the amplitude of excitatory postsynaptic currents (EPSCs) and increases the amplitude of inhibitory postsynaptic currents (IPSCs), with no change in their frequency. This is surprising because as a presynaptic protein, α-synuclein would be expected to influence event frequency. A change in amplitude is generally considered to reflect a change in postsynaptic receptors, where there is relatively little synuclein. Alternatively, a change in amplitude might reflect altered synaptic vesicle filling with neurotransmitter and synuclein might affect leakage from synaptic vesicles but complete absence of synuclein has not previously been shown to affect either of these properties. The authors also examine evoked release in hippocampal slices and find a modest effect on paired-pulse ratio and a larger effect on synaptic depression, parameters more clearly associated with presynaptic function but again without major change in synuclein KO mice^16^.

In summary, the authors provide clear evidence for physiological regulation of α-synuclein phosphorylation at Ser-129 by neural activity, indicating a role beyond Lewy pathology. In addition to Ca^++^ acting through calcineurin and Plk2, membrane association appears to regulate pSer-129 but pSer-129 does not apparently influence membrane association. The modification also appears to affect neurotransmission although the mechanism remains unclear. Perhaps most interesting, the results suggest an important regulatory role for the less conserved α-synuclein C-terminus. The point mutations that cause PD occur within a small region in the N-terminal membrane-binding repeats, but regulation at the C-terminus may control the function of synuclein in degeneration as well as physiology.

## Data Availability

Data sharing not applicable to this article as no datasets were generated or analysed in the preparation of this Comment.
